# Crystal structure of (1,4,7,10,13,16-hexa­oxa­cycloocta­decane-κ^6^*O*)potassium-μ-oxalato-tri­phenylstannate(IV), the first reported 18-crown-6-stabilized potassium salt of tri­phenyl­oxalatostannate

**DOI:** 10.1107/S2056989024007758

**Published:** 2024-08-13

**Authors:** Xueqing Song, William Li, Yolanda Torres, Tazena Greaves

**Affiliations:** a University of the District of Columbia, Division of Sciences and Mathematics 4200 Connecticut Avenue NW Washington DC 20008 USA; Universidad de la Repüblica, Uruguay

**Keywords:** crystal Structure, tri­phenyl­stannate, oxalato, 18-crown-6, potassium

## Abstract

The title complex, (1,4,7,10,13,16-hexa­oxa­cyclo­octa­decane-1κ^6^*O*)(μ-oxalato-1κ^2^*O*^1^,*O*^2^:2κ^2^*O*^1′^,*O*^2′^)triphenyl-2κ^3^*C*-potassium(I)tin(IV), [KSn(C_6_H_5_)_3_(C_2_O_4_)(C_12_H_24_O_6_)] or K[18-Crown-6][(C_6_H_5_)_3_SnO_4_C_2_], was synthesized. The complex consists of a potassium cation coordinated to the six oxygen atoms of a crown ether mol­ecule and the two oxygen atoms of the oxalatotri­phenyl­stannate anion. It crystallizes in the monoclinic crystal system within the space group *P*2_1_. The tin atom is coordinated by one chelating oxalate ligand and three phenyl groups, forming a *cis*-trigonal–bipyramidal geometry around the tin atom. The cations and anions form ion pairs, linked through carbonyl coordination to the potassium atoms. The crystal structure features C—H⋯O hydrogen bonds between the oxygen atoms of the oxalate group and the hydrogen atoms of the phenyl groups, resulting in an infinite chain structure extending along *a*-axis direction. The primary inter-chain inter­actions are van der Waals forces.

## Abstract 2

The single-crystal X-ray structure of [(18-crown-6)K][SnPh_3_(ox)] (ox = C_2_O_4_^2−^) is reported. Integrity between neighboring mol­ecules in the solid state is maintained by an array of C—H⋯O hydrogen bonds and C—H⋯π inter­actions.

## 1. Chemical context

Organotin carboxyl­ates are one of the most significant classes of compounds, valued not only for their theoretical and structural properties but also for their industrial and agricultural applications (Zuckermann *et al.*, 1976 ▸). Organotin(IV) carboxyl­ates are particularly notable for their diverse and important biological activities, serving as anti­cancer, anti­viral, anti­bacterial, and anti­fungal agents, as well as wood preservatives and pesticides (Davies & Smith, 1980 ▸; Smith *et al.*, 1978 ▸; Thayer *et al.*, 1984 ▸; Blunden *et al.*, 1985 ▸; Evans & Karpel, 1985 ▸; Angham *et al.*, 2019 ▸; Talebi *et al.*, 2023 ▸). Metal complexes of di­carb­oxy­lic acids, such as oxalic acid, have garnered significant inter­est due to their promising magnetic and electrochemical properties. The appeal of oxalate-based coordination compounds lies in their high structural diversity, attributed to the oxalate ligand’s ability to adopt 17 different coordination modes and function as a mono-, bi-, tri-, or tetra­dentate ligand (Krishnamurty & Harris, 1961 ▸; Rao *et al.*, 2004 ▸). This results in a vast, yet largely unexplored, compositional area. Notably, there are very few reports of organotin complexes of oxalic acid in the literature.

The author has been inter­ested in designing and preparing ionic organotin complexes to improve aqueous solubility through ionization. Since the pioneering work of Pedersen (Pedersen, 1988 ▸; Izatt, 2017 ▸), crown ethers and their complexes with metal cations have attracted considerable attention. Their remarkable selectivity on metal cations, especially alkali and alkaline earth metal cations, is a topic of fundamental inter­est in both coordination chemistry and biological chemistry (Bajaj *et al.*, 1988 ▸; Hay & Rustad, 1994 ▸; Lehn *et al.*, 1988 ▸; Lee *et al.*, 1996 ▸). Literature reports show that crown ethers can be utilized in solid-solid and solid-liquid processes to capture alkali metal and ammonium cations in extended hydrogen-bonded networks formed by inorganic acid anions, such as hydrogen sulfate and di-hydrogen phosphate, as well as organic acid anions (Braga *et al.*, 2005 ▸, 2007 ▸, 2008 ▸, 2009 ▸). In this context, we present and discuss the crystal structure of a crown ether-stabilized potassium salt of oxalatotri­phenyl­stannate, **1**.
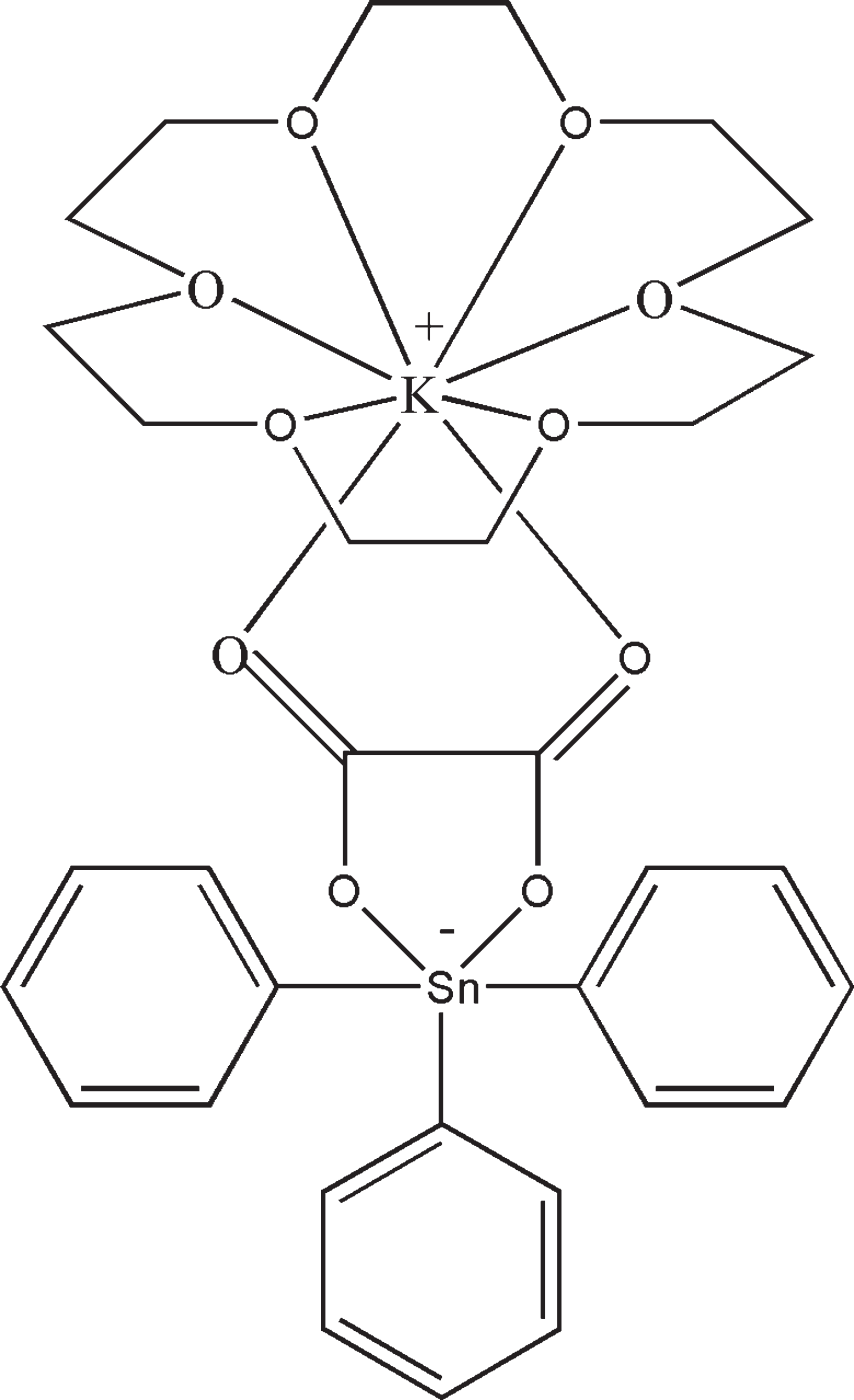


## 2. Structural commentary

**Figure 1:**
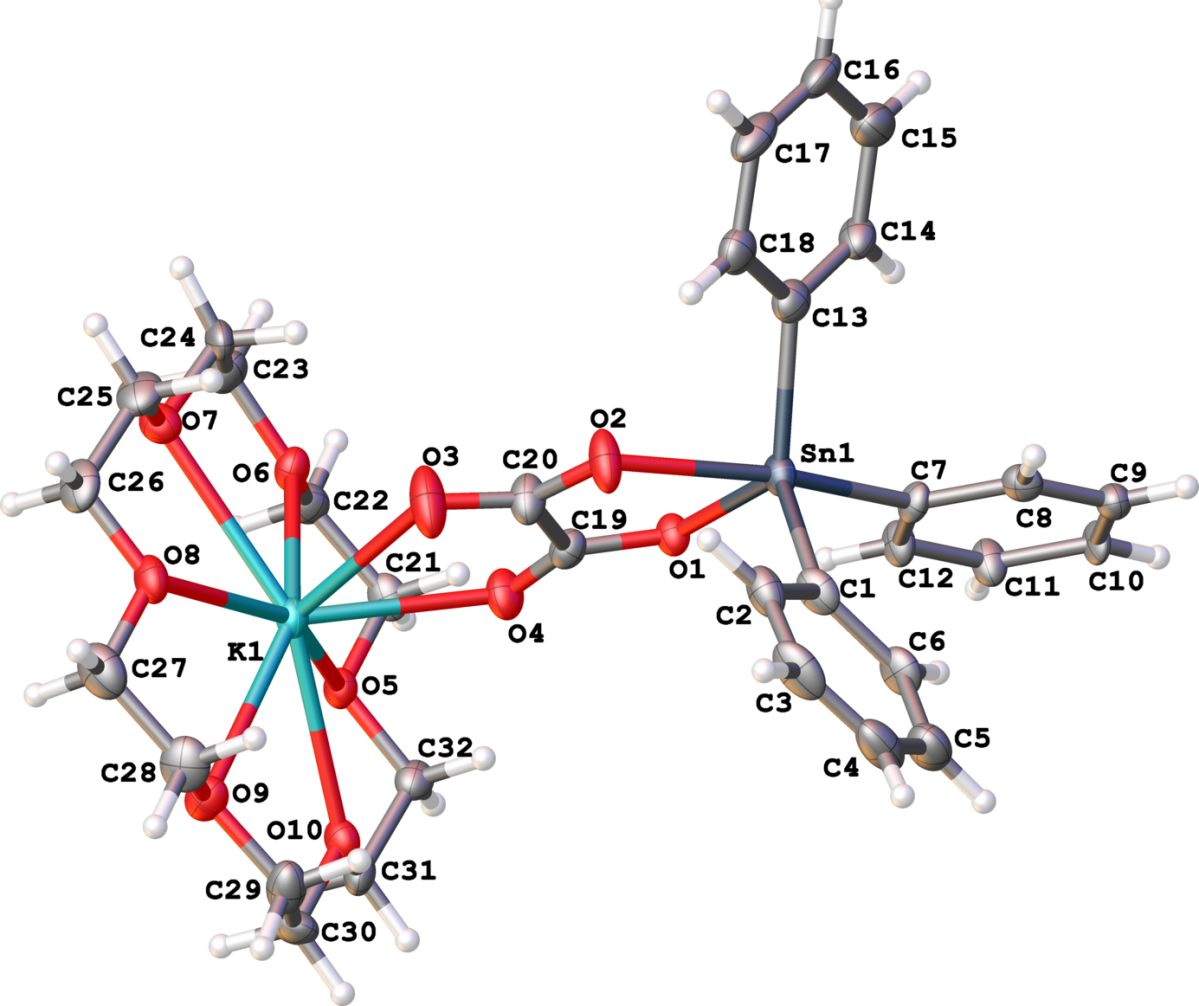
The asymmetric unit and mol­ecular structure of crystal [(18-crown-6)K][SnPh_3_(ox)] (**1**) with anisotropic displacement ellipsoids set to the 50% probability level.

The stannate anionic unit of the title compound **1** features a *cis*-tbp [Ph_3_Snox]^−^ anion, with Sn1—O1 measuring 2.071 (5) Å and Sn1—O2 measuring 2.290 (6) Å, and an O1—Sn1—O2 bond angle of 73.4 (2)° (Fig. 1 ▸). This anion is coordinated *via* its two oxalate carbonyl groups (O3 and O4) to a K[18-crown-6] cation, with K1—O3 at 2.785 (7) Å, K1—O4 at 2.654 (6) Å, and an O3—K1—O4 bond angle of 61.3 (2)° (Fig. 1 ▸). The oxalate acts as a bidentate ligand to both tin and potassium, forming two five-membered chelate rings that are coplanar, with a dihedral angle of approximately 0°.

In the [Ph_3_Snox]^−^ portion, the axial Sn-O bond [Sn1—O2 at 2.290 (6) Å] is significantly longer than the equatorial Sn-O bond [Sn1—O1 at 2.071 (5) Å]. The bite angle of 73.4 (2)° is similar to those found in other chelated oxalato tri­phenyl­stannates (Ng *et al.*, 1992 ▸; Ng & Kumar Das, 1993 ▸; Ng, 1996 ▸). The axial Sn—C bond [Sn1—C7 at 2.188 (7) Å] is somewhat longer than the equatorial Sn—C bonds [Sn1—C1 at 2.136 (7) Å and Sn1—C13 at 2.138 (7) Å]. The axial structure is notably bent, with an O2—Sn1—C7 angle of 160.8 (3)°, and the Sn atom is displaced out of the equatorial plane [Σ angles at Sn = 354.9 (3)°] towards the axial C7 atom by 0.119 Å.

The oxalate group in the [Ph_3_Snox]^−^ ion consists of two similar carboxyl­ate (–COO^−^) entities. Both bind to the Sn1 and K1 atoms, with slightly different C—O bond lengths: those bonded to tin [C—O = 1.266 (11) and 1.303 (10) Å] are slightly longer than those bonded to potassium [C—O = 1.233 (11) and 1.202 (10) Å]. The two negative charges appear to be delocalized over the four oxygen atoms in the oxalate group.

In the [K(18-crown-6)]^+^ complex cation, the potassium atom deviates by 0.614 Å from the root-mean-square plane of the six oxygen atoms in the 18-crown-6 ligand towards the oxalate group. This deviation is due to the coordination of two oxygens from the oxalate group. A similar observation has been reported in the literature (Gjikaj *et al.*, 2005 ▸; Liebing *et al.*, 2016 ▸; Sellin & Malischewski, 2019 ▸) when potassium has axial coordination to other heteroatoms. The K—O bond lengths with the 18-crown-6 ligand range from 2.802 (6) to 2.976 (6) Å, which are slightly longer than those reported for other [K(18-crown-6)]^+^ complexes in the literature. The increased average K—O bond length with 18-crown-6 is attributed to the strong coordination with the oxalate group, where the two K—O bond lengths with the oxalate group are 2.654 (6) Å and 2.785 (7) Å. The coordination number of the K^+^ cation is 8. The coordination polyhedron of the potassium cation can be described as a distorted hexa­gonal pyramid with a bifurcated vertex at the O3 and O4 atoms.

## 3. Supra­molecular features

**Figure 2:**
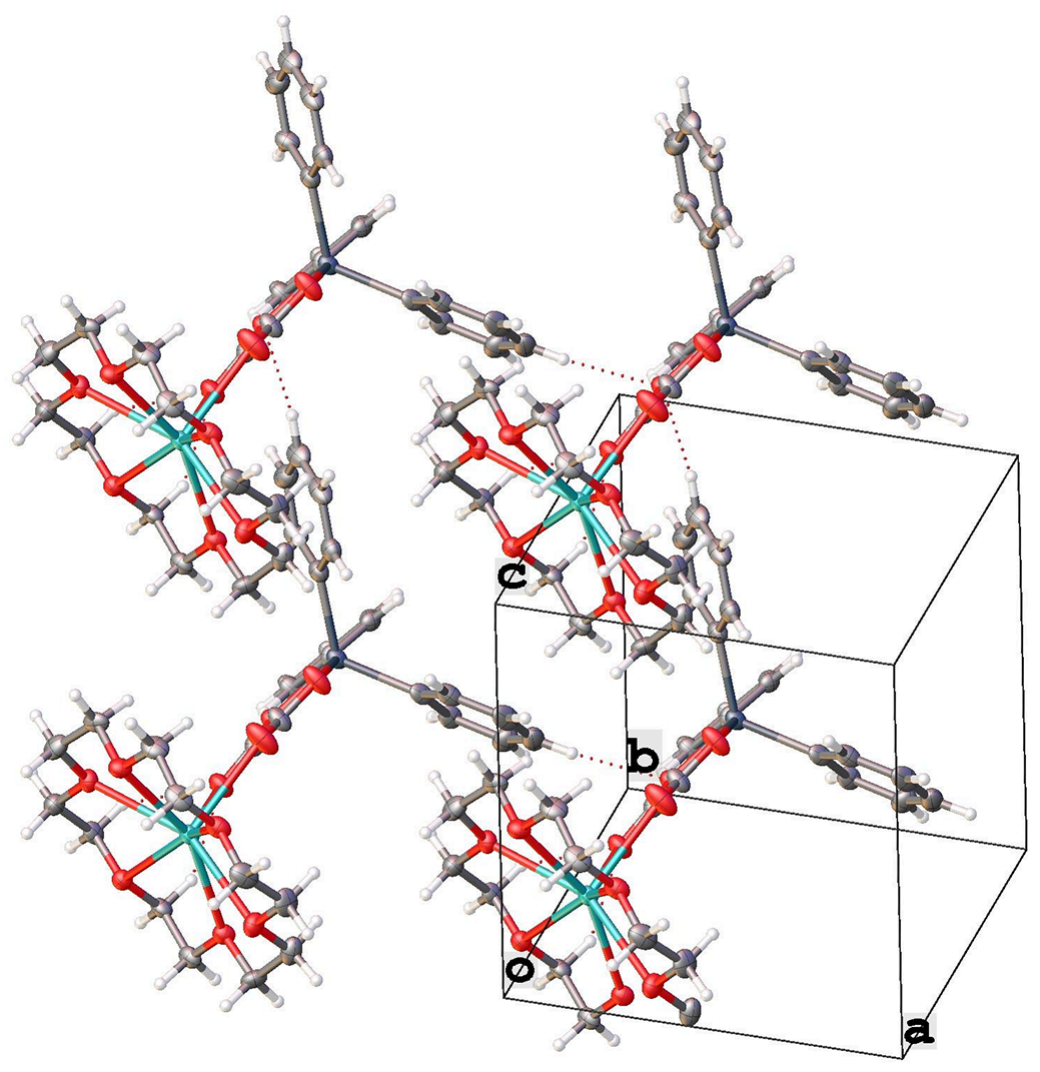
Crystal packing in the crystal structure showing C—H⋯O hydrogen bonds, denoted by dashed lines, between neighboring mol­ecules.

**Figure 3:**
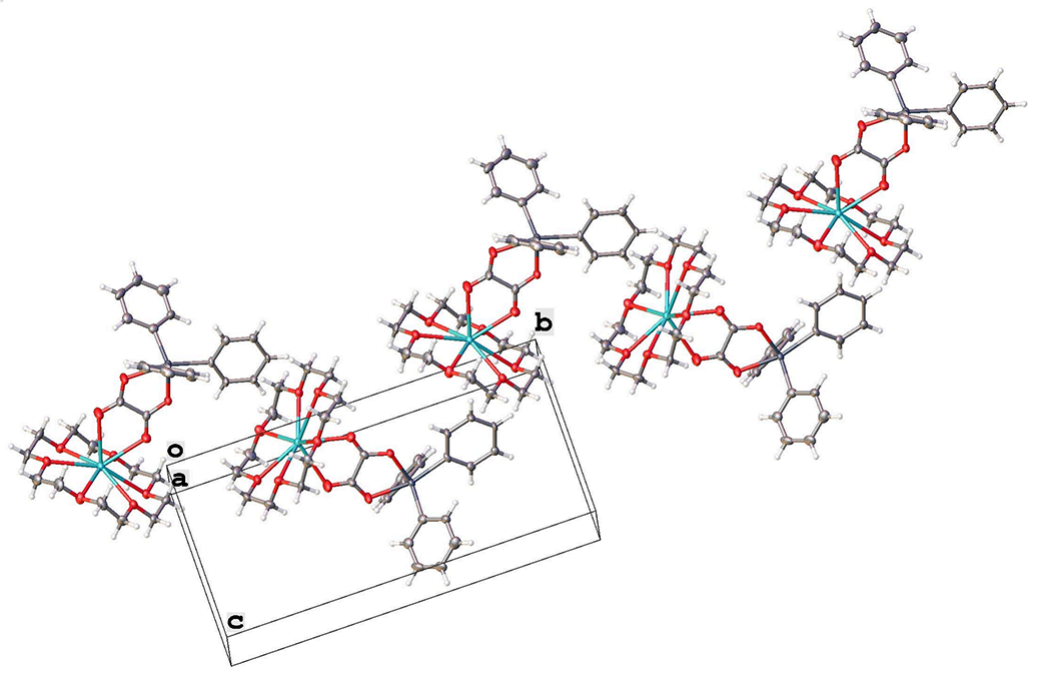
Partial packing plot of **1** along the *b* axis showing the 1-D chain formed through C—H⋯O hydrogen bonding.

The title complex **1** exhibits a supra­molecular structure that is consolidated by two weak inter­molecular hydrogen bonds: C4—H4⋯O1 and C16—H16⋯O1, with C⋯O distances of 3.360 (11) and 3.332 (9) Å, respectively (Fig. 2 ▸; symmetry codes as in Table 1 ▸). These inter­molecular hydrogen bonds result in the formation of a ‘shoulder-to-shoulder’ arrangement of the complex mol­ecules, creating a supra­molecular layer parallel to the (001) plane, as depicted in Figs. 3 ▸ and 4 ▸.

In supra­molecular chemistry, weak hydrogen bonds such as C—H⋯π and π–π inter­actions play significant roles in the structural integrity of crystal structures (Meyer *et al.*, 2003 ▸; Nishio, 2004 ▸). The effectiveness of these inter­actions is primarily influenced by the distance between the hydrogen atom of the C—H bond and the plane of the aromatic ring, which should be less than 2.9 Å (the combined van der Waals radii), and the C—H⋯π access angles, ideally ranging from 140 to 180° (Takahashi *et al.*, 2001 ▸).

In the crystal under investigation, relatively weak C—H⋯π inter­actions are observed. The analysis of these inter­actions reveals C—H⋯centroid phenyl distances of 2.936 and 2.937 Å (for H26*A*⋯C11) and distances of 2.937 and 2.949 Å (for H27*B*⋯C13). The corresponding access angles are 158° for C1 and 162° for C13. Despite the parallel alignment of phenyl groups (C7–C12) along the (101) direction, significant π–π inter­actions are absent. This is attributed to a large separation distance of 7.006 Å between the planes, which greatly exceeds the critical distance of 4 Å, and an inter-centroid distance of 9.406 Å, surpassing the 6 Å threshold (Ninković *et al.*, 2011 ▸).

## 4. Database survey

A survey of the Cambridge Structural Database (CSD; Groom *et al.*, 2016 ▸; Conquest version 2024.1.0, Build 401958; Bruno *et al.*, 2002 ▸) reveals thirteen reports of oxalatotri­phenyl­stannate compounds with ammonium ions as counter-ions. Examples include di-*iso*-propyl­ammonium (Ng & Hook, 1999 ▸), di-*cyclo*-hexyl­ammonium (Ng & Rae, 2000 ▸), di­benzyl­ammonium (Gueye *et al.*, 2012 ▸), and di-*iso*-butyl­ammonium (Thorpe *et al.*, 2013 ▸). A further search for metal salts of tri­phenyl­stannate came out with one hit, in which a sodium salt of tri­phenyl­stannate named sodium bis­[2-(3′,6′,9′-trioxadec­yl)-1,2-dicarba-closododeca­boane-1-carboxyl­ato]tri­phenyl­stannate was reported (Bregadze *et al.*, 2004 ▸). In this reported stannate, the sodium ion is stabilized by coordination to the carbonyl oxygen and five oxygen atoms of trioxadecyl substituents. In contrast, in the title compound **1**, the potassium salt of tri­phenyl­stannate is described for the first time, where the potassium ion is primarily stabilized through coordination to 18-crown-6.

## 5. Synthesis and crystallization

The title coordination complex of tri­phenyl­tin was synthesized by reacting 1 mmol of oxalic acid, 1 mmol of potassium bicarbonate, 1 mmol of 18-crown-6, and 1 mmol of tri­phenyl­tin hydroxide in 30 mL of ethanol. The mixture was refluxed at 373 K with stirring for 1 h. The resulting solution, which was slightly cloudy, was filtered to yield a clear ethanol solution. This filtrate was then allowed to evaporate slowly at 300 K over the course of one week, resulting in colorless crystals suitable for X-ray diffraction analysis.

## 6. Refinement

Crystal data, data collection and structure refinement details are summarized in Table 2 ▸. The H atoms in compound **1** were placed in geometrically idealized positions and constrained to ride on their parent atoms, with C—H distances of 0.93 Å (ring H atoms) and 0.97 Å (methyl­ene H atoms), and N—H distances of 0.98 Å, with *U*_iso_(H) values of 1.2*U*_eq_ of the parent atoms. Reflections were merged by *SHELXL* according to the crystal class for the calculation of statistics and refinement. The Friedel fraction is defined as the number of unique Friedel pairs measured divided by the number that would be possible theoretically, ignoring centric projections and systematic absences. Ther crystal studied was refined as a two-component twin. Completeness statistics refer to single and composite reflections containing twin component 1 only.

## Supplementary Material

Crystal structure: contains datablock(s) I. DOI: 10.1107/S2056989024007758/ny2007sup1.cif

Supporting information file. DOI: 10.1107/S2056989024007758/ny2007Isup3.mol

Structure factors: contains datablock(s) I. DOI: 10.1107/S2056989024007758/ny2007Isup4.hkl

CCDC reference: 2375984

Additional supporting information:  crystallographic information; 3D view; checkCIF report

## Figures and Tables

**Figure 4:**
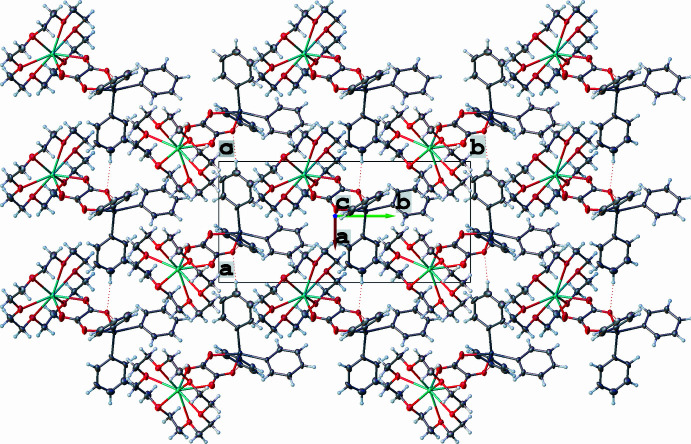
Packing plot of **1** viewed approximately along [001] showing a layer of mol­ecules perpendicular to the *c* axis.

**Table 1:** Hydrogen-bond geometry (Å, °)

*D*—H⋯*A*	*D*—H	H⋯*A*	*D*⋯*A*	*D*—H⋯*A*
C4—H4⋯O1^i^	0.95	2.42	3.360 (11)	172
C16—H16⋯O1^ii^	0.95	2.41	3.332 (9)	165

Symmetry codes: (i) 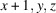; (ii) 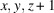.

**Table 2:** Experimental details

Crystal data	
Chemical formula	[KSn(C_6_H_5_)_3_(C_2_O_4_)(C_12_H_24_O_6_)]
*M*_r_	741.42
Crystal system, space group	Monoclinic, *P*2_1_
Temperature (K)	100
*a*, *b*, *c* (Å)	9.4060 (3), 19.3779 (4), 9.4225 (3)
β (°)	97.925 (2)
*V* (Å^3^)	1701.02 (8)
*Z*	2
Radiation type	Mo *K*α
μ (mm^−1^)	0.93
Crystal size (mm)	0.06 × 0.06 × 0.03 × 0.02 (radius)

Data collection	
Diffractometer	Rigaku XtaLAB Synergy-S dual wavelength Mo/Cu
Absorption correction	Multi-scan (*CrysAlis PRO*; Rigaku OD, 2022 ▸)
*T*_min_, *T*_max_	0.913, 1.000
No. of measured, independent and observed [*I* > 2σ(*I*)] reflections	15412, 7113, 6792
*R*_int_	0.040
(sin θ/λ)_max_ (Å^−1^)	0.649

Refinement	
*R*[*F*^2^ > 2σ(*F*^2^)], *wR*(*F*^2^), *S*	0.033, 0.105, 1.08
No. of reflections	7113
No. of parameters	398
No. of restraints	1
H-atom treatment	H-atom parameters constrained
Δρ_max_, Δρ_min_ (e Å^−3^)	1.28, −0.52
Absolute structure	Flack *x* determined using 2807 quotients [(*I*^+^)−(*I*^−^)]/[(*I*^+^)+(*I*^−^)] (Parsons *et al.*, 2013 ▸)
Absolute structure parameter	−0.08 (3)

Computer programs: *CrysAlis PRO* (Rigaku OD, 2022 ▸), *SHELXT* (Sheldrick, 2015a) ▸, *SHELXL2018/3* (Sheldrick, 2015*b* ▸) and *OLEX2* (Dolomanov *et al.*, 2009 ▸).

## supplementary crystallographic information

### (1,4,7,10,13,16-Hexaoxacyclooctadecane-1κ6O)(µ-oxalato-1κ2O1,O2:2κ2O1',O2')triphenyl-2κ3C-potassium(I)tin(IV) Crystal data

**Table:**

[KSn(C_6_H_5_)_3_(C_2_O_4_)(C_12_H_24_O_6_)]	*F*(000) = 760
*M**_r_* = 741.42	*D*_x_ = 1.448 Mg m^−^^3^
Monoclinic, *P*2_1_	Mo *K*α radiation, λ = 0.71073 Å
*a* = 9.4060 (3) Å	Cell parameters from 9822 reflections
*b* = 19.3779 (4) Å	θ = 2.9–31.1°
*c* = 9.4225 (3) Å	µ = 0.93 mm^−^^1^
β = 97.925 (2)°	*T* = 100 K
*V* = 1701.02 (8) Å^3^	Block, colorless
*Z* = 2	0.06 × 0.06 × 0.03 × 0.02 (radius) mm

### (1,4,7,10,13,16-Hexaoxacyclooctadecane-1κ6O)(µ-oxalato-1κ2O1,O2:2κ2O1',O2')triphenyl-2κ3C-potassium(I)tin(IV) Data collection

**Table:**

Rigaku XtaLAB Synergy-S dual wavelength Mo/Cu diffractometer	7113 independent reflections
Radiation source: microfocus sealed X-ray tube, Rigaku PhotonJet-S	6792 reflections with *I* > 2σ(*I*)
Mirror optics monochromator	*R*_int_ = 0.040
Detector resolution: 10.0000 pixels mm^-1^	θ_max_ = 27.5°, θ_min_ = 2.9°
ω scans	*h* = −12→11
Absorption correction: multi-scan (CrysAlis PRO; Rigaku OD, 2022)	*k* = −23→25
*T*_min_ = 0.913, *T*_max_ = 1.000	*l* = −11→10
15412 measured reflections	

### (1,4,7,10,13,16-Hexaoxacyclooctadecane-1κ6O)(µ-oxalato-1κ2O1,O2:2κ2O1',O2')triphenyl-2κ3C-potassium(I)tin(IV) Refinement

**Table:**

Refinement on *F*^2^	Hydrogen site location: inferred from neighbouring sites
Least-squares matrix: full	H-atom parameters constrained
*R*[*F*^2^ > 2σ(*F*^2^)] = 0.033	*w* = 1/[σ^2^(*F*_o_^2^) + (0.0708*P*)^2^] where *P* = (*F*_o_^2^ + 2*F*_c_^2^)/3
*wR*(*F*^2^) = 0.105	(Δ/σ)_max_ < 0.001
*S* = 1.08	Δρ_max_ = 1.28 e Å^−^^3^
7113 reflections	Δρ_min_ = −0.52 e Å^−^^3^
398 parameters	Absolute structure: Flack *x* determined using 2807 quotients [(*I*^+^)-(*I*^-^)]/[(*I*^+^)+(*I*^-^)] (Parsons *et al.*, 2013)
1 restraint	Absolute structure parameter: −0.08 (3)
Primary atom site location: structure-invariant direct methods	

### (1,4,7,10,13,16-Hexaoxacyclooctadecane-1κ6O)(µ-oxalato-1κ2O1,O2:2κ2O1',O2')triphenyl-2κ3C-potassium(I)tin(IV) Special details

**Table:**

Geometry. All esds (except the esd in the dihedral angle between two l.s. planes) are estimated using the full covariance matrix. The cell esds are taken into account individually in the estimation of esds in distances, angles and torsion angles; correlations between esds in cell parameters are only used when they are defined by crystal symmetry. An approximate (isotropic) treatment of cell esds is used for estimating esds involving l.s. planes.
Refinement. Refined as a 2-component twin.

### (1,4,7,10,13,16-Hexaoxacyclooctadecane-1κ6O)(µ-oxalato-1κ2O1,O2:2κ2O1',O2')triphenyl-2κ3C-potassium(I)tin(IV) Fractional atomic coordinates and isotropic or equivalent isotropic displacement parameters (Å^2^)

**Table:**

	*x*	*y*	*z*	*U*_iso_*/*U*_eq_	
Sn1	0.41008 (5)	0.58376 (2)	0.45935 (5)	0.02209 (12)	
O1	0.2517 (6)	0.5552 (3)	0.2950 (6)	0.0223 (10)	
O2	0.3969 (8)	0.4658 (3)	0.4651 (8)	0.0369 (16)	
O3	0.2847 (9)	0.3760 (4)	0.3470 (9)	0.0484 (19)	
O4	0.1482 (7)	0.4720 (3)	0.1562 (7)	0.0312 (14)	
C1	0.6233 (8)	0.5741 (5)	0.4077 (8)	0.0256 (19)	
C2	0.7115 (10)	0.5176 (4)	0.4492 (9)	0.0306 (18)	
H2	0.677139	0.480378	0.500564	0.037*	
C3	0.8536 (11)	0.5166 (5)	0.4135 (11)	0.039 (2)	
H3	0.913985	0.478341	0.441871	0.047*	
C4	0.9046 (10)	0.5691 (5)	0.3399 (10)	0.040 (3)	
H4	1.000617	0.568146	0.319164	0.048*	
C5	0.8163 (11)	0.6238 (5)	0.2954 (12)	0.038 (2)	
H5	0.850759	0.659627	0.240293	0.045*	
C6	0.6788 (9)	0.6274 (4)	0.3295 (10)	0.0280 (17)	
H6	0.620610	0.666331	0.300060	0.034*	
C7	0.3592 (8)	0.6911 (4)	0.4013 (9)	0.0211 (15)	
C8	0.4450 (8)	0.7424 (4)	0.4748 (8)	0.0237 (15)	
H8	0.519593	0.729495	0.548454	0.028*	
C9	0.4225 (8)	0.8126 (4)	0.4413 (8)	0.0232 (14)	
H9	0.480264	0.846932	0.492962	0.028*	
C10	0.3137 (8)	0.8314 (4)	0.3306 (8)	0.0225 (15)	
H10	0.298609	0.878641	0.305833	0.027*	
C11	0.2278 (9)	0.7806 (4)	0.2571 (9)	0.0265 (16)	
H11	0.152693	0.793270	0.183718	0.032*	
C12	0.2525 (9)	0.7106 (4)	0.2917 (9)	0.0246 (16)	
H12	0.195505	0.676187	0.239377	0.030*	
C13	0.3708 (7)	0.5795 (7)	0.6773 (8)	0.0273 (15)	
C14	0.2974 (10)	0.6343 (5)	0.7297 (9)	0.0282 (17)	
H14	0.262058	0.670815	0.667257	0.034*	
C15	0.2754 (11)	0.6360 (5)	0.8732 (11)	0.038 (2)	
H15	0.226304	0.673758	0.908593	0.046*	
C16	0.3256 (8)	0.5824 (8)	0.9638 (8)	0.0354 (17)	
H16	0.310981	0.583366	1.061508	0.042*	
C17	0.3963 (10)	0.5280 (6)	0.9124 (10)	0.038 (2)	
H17	0.429710	0.491448	0.975628	0.045*	
C18	0.4207 (9)	0.5247 (5)	0.7686 (9)	0.0307 (18)	
H18	0.469574	0.486627	0.734140	0.037*	
C19	0.2277 (8)	0.4908 (4)	0.2599 (9)	0.0227 (15)	
C20	0.3101 (10)	0.4379 (4)	0.3672 (10)	0.0287 (16)	
K1	0.10867 (17)	0.33895 (8)	0.09631 (17)	0.0219 (3)	
O5	−0.0911 (6)	0.4275 (3)	−0.0971 (6)	0.0229 (11)	
O6	−0.1562 (6)	0.3828 (3)	0.1682 (6)	0.0248 (12)	
O7	−0.0356 (7)	0.2587 (4)	0.2852 (7)	0.0275 (15)	
O8	0.2230 (7)	0.2108 (3)	0.2005 (7)	0.0292 (12)	
O9	0.2918 (8)	0.2593 (4)	−0.0621 (8)	0.0294 (16)	
O10	0.1753 (6)	0.3845 (3)	−0.1714 (6)	0.0250 (12)	
C21	−0.1744 (9)	0.4682 (4)	−0.0123 (9)	0.0258 (16)	
H21A	−0.241853	0.497889	−0.075143	0.031*	
H21B	−0.110542	0.498315	0.053253	0.031*	
C22	−0.2558 (11)	0.4209 (6)	0.0720 (11)	0.029 (2)	
H22A	−0.319867	0.447871	0.126044	0.035*	
H22B	−0.315664	0.389015	0.006764	0.035*	
C23	−0.2230 (12)	0.3420 (6)	0.2648 (12)	0.035 (2)	
H23A	−0.286849	0.307414	0.211248	0.042*	
H23B	−0.281701	0.371518	0.319785	0.042*	
C24	−0.1089 (10)	0.3064 (4)	0.3645 (9)	0.0276 (17)	
H24A	−0.040179	0.340835	0.411697	0.033*	
H24B	−0.152538	0.281731	0.439726	0.033*	
C25	0.0662 (11)	0.2172 (5)	0.3752 (10)	0.035 (2)	
H25A	0.016438	0.190038	0.442444	0.042*	
H25B	0.137948	0.247248	0.431986	0.042*	
C26	0.1389 (11)	0.1701 (4)	0.2836 (10)	0.0339 (19)	
H26A	0.201246	0.137366	0.344216	0.041*	
H26B	0.066709	0.143313	0.219869	0.041*	
C27	0.2983 (11)	0.1707 (5)	0.1108 (11)	0.039 (2)	
H27A	0.229728	0.144494	0.041901	0.047*	
H27B	0.361344	0.137377	0.168962	0.047*	
C28	0.3872 (10)	0.2180 (5)	0.0311 (11)	0.036 (2)	
H28A	0.449335	0.247442	0.099552	0.044*	
H28B	0.449059	0.190529	−0.024455	0.044*	
C29	0.3694 (10)	0.3065 (5)	−0.1418 (11)	0.035 (2)	
H29A	0.439272	0.280956	−0.191382	0.043*	
H29B	0.422566	0.340498	−0.076177	0.043*	
C30	0.2618 (11)	0.3427 (5)	−0.2491 (10)	0.032 (2)	
H30A	0.311547	0.371631	−0.313522	0.038*	
H30B	0.201563	0.308548	−0.307951	0.038*	
C31	0.0763 (10)	0.4239 (5)	−0.2660 (10)	0.027 (2)	
H31A	0.010250	0.392884	−0.326933	0.032*	
H31B	0.128454	0.452511	−0.329063	0.032*	
C32	−0.0073 (9)	0.4696 (4)	−0.1780 (8)	0.0253 (16)	
H32A	0.059398	0.498412	−0.112634	0.030*	
H32B	−0.070902	0.500531	−0.241853	0.030*	

### (1,4,7,10,13,16-Hexaoxacyclooctadecane-1κ6O)(µ-oxalato-1κ2O1,O2:2κ2O1',O2')triphenyl-2κ3C-potassium(I)tin(IV) Atomic displacement parameters (Å^2^)

**Table:**

	*U*^11^	*U*^22^	*U*^33^	*U*^12^	*U*^13^	*U*^23^
Sn1	0.0258 (2)	0.01459 (18)	0.0242 (2)	0.0024 (3)	−0.00227 (13)	0.0011 (3)
O1	0.026 (3)	0.016 (2)	0.024 (3)	0.002 (2)	−0.0018 (19)	0.001 (2)
O2	0.045 (4)	0.016 (3)	0.043 (4)	−0.001 (3)	−0.018 (3)	−0.001 (3)
O3	0.061 (5)	0.022 (3)	0.053 (5)	−0.004 (3)	−0.024 (3)	−0.002 (3)
O4	0.034 (3)	0.025 (3)	0.031 (3)	−0.003 (2)	−0.007 (2)	−0.002 (2)
C1	0.027 (3)	0.021 (5)	0.027 (3)	0.014 (3)	−0.005 (3)	−0.001 (3)
C2	0.038 (5)	0.020 (4)	0.030 (5)	0.006 (3)	−0.007 (3)	−0.003 (3)
C3	0.036 (5)	0.034 (5)	0.043 (5)	0.023 (4)	−0.008 (4)	−0.012 (4)
C4	0.030 (4)	0.046 (8)	0.044 (5)	0.001 (4)	0.005 (3)	−0.022 (5)
C5	0.039 (5)	0.032 (5)	0.043 (6)	0.002 (4)	0.013 (4)	−0.010 (4)
C6	0.028 (4)	0.020 (4)	0.035 (5)	0.012 (3)	0.000 (3)	−0.003 (3)
C7	0.022 (4)	0.012 (3)	0.030 (5)	0.002 (3)	0.004 (3)	0.005 (3)
C8	0.024 (4)	0.023 (4)	0.024 (4)	0.006 (3)	0.005 (3)	0.001 (3)
C9	0.028 (4)	0.020 (3)	0.023 (4)	−0.001 (3)	0.006 (3)	−0.001 (3)
C10	0.032 (4)	0.008 (3)	0.027 (4)	0.002 (3)	0.000 (3)	0.002 (3)
C11	0.035 (4)	0.018 (3)	0.024 (4)	0.002 (3)	−0.008 (3)	0.002 (3)
C12	0.030 (4)	0.013 (4)	0.028 (5)	0.004 (3)	−0.003 (3)	0.002 (3)
C13	0.026 (3)	0.028 (4)	0.027 (3)	−0.005 (5)	−0.001 (2)	0.001 (5)
C14	0.038 (5)	0.022 (4)	0.023 (4)	−0.004 (3)	−0.001 (3)	0.004 (3)
C15	0.039 (5)	0.040 (5)	0.038 (5)	−0.010 (4)	0.012 (4)	−0.005 (4)
C16	0.039 (4)	0.034 (4)	0.032 (4)	−0.016 (6)	0.004 (3)	0.009 (6)
C17	0.038 (5)	0.041 (5)	0.030 (4)	−0.015 (4)	−0.006 (3)	0.017 (4)
C18	0.027 (4)	0.029 (4)	0.035 (5)	−0.008 (3)	0.001 (3)	0.000 (4)
C19	0.021 (4)	0.020 (4)	0.027 (4)	0.001 (3)	0.003 (3)	0.004 (3)
C20	0.032 (5)	0.020 (4)	0.029 (5)	−0.001 (3)	−0.010 (3)	0.004 (3)
K1	0.0241 (7)	0.0168 (7)	0.0240 (8)	−0.0005 (5)	0.0001 (5)	−0.0004 (5)
O5	0.026 (3)	0.017 (2)	0.026 (3)	0.000 (2)	0.0027 (19)	−0.0001 (19)
O6	0.022 (3)	0.020 (3)	0.032 (3)	−0.002 (2)	0.003 (2)	0.001 (2)
O7	0.033 (4)	0.026 (3)	0.023 (3)	0.003 (3)	0.002 (2)	0.001 (3)
O8	0.039 (3)	0.017 (3)	0.030 (3)	0.000 (2)	−0.002 (2)	−0.001 (2)
O9	0.026 (3)	0.026 (3)	0.037 (4)	0.004 (3)	0.007 (3)	0.003 (3)
O10	0.030 (3)	0.018 (3)	0.026 (3)	0.002 (2)	0.002 (2)	−0.002 (2)
C21	0.023 (4)	0.021 (4)	0.031 (4)	0.009 (3)	−0.004 (3)	−0.001 (3)
C22	0.027 (5)	0.032 (5)	0.029 (5)	0.000 (4)	0.006 (4)	−0.002 (4)
C23	0.039 (5)	0.028 (5)	0.042 (5)	−0.007 (4)	0.018 (4)	−0.001 (4)
C24	0.041 (5)	0.015 (4)	0.027 (4)	−0.006 (3)	0.007 (3)	−0.004 (3)
C25	0.048 (5)	0.031 (4)	0.025 (4)	−0.002 (4)	0.001 (4)	0.012 (4)
C26	0.045 (5)	0.018 (4)	0.035 (5)	0.003 (4)	−0.005 (4)	0.011 (3)
C27	0.047 (6)	0.029 (5)	0.040 (5)	0.014 (4)	−0.004 (4)	−0.002 (4)
C28	0.028 (4)	0.030 (4)	0.049 (6)	0.014 (4)	0.001 (4)	0.003 (4)
C29	0.029 (4)	0.025 (4)	0.054 (6)	0.000 (4)	0.011 (4)	−0.005 (4)
C30	0.041 (5)	0.031 (5)	0.025 (4)	0.005 (4)	0.013 (4)	−0.003 (4)
C31	0.032 (5)	0.019 (4)	0.027 (5)	−0.007 (3)	0.000 (4)	0.001 (3)
C32	0.028 (4)	0.026 (4)	0.020 (4)	−0.003 (3)	−0.002 (3)	0.003 (3)

### (1,4,7,10,13,16-Hexaoxacyclooctadecane-1κ6O)(µ-oxalato-1κ2O1,O2:2κ2O1',O2')triphenyl-2κ3C-potassium(I)tin(IV) Geometric parameters (Å, º)

**Table:**

Sn1—O1	2.071 (5)	K1—O7	2.845 (7)
Sn1—O2	2.290 (6)	K1—O8	2.827 (6)
Sn1—C1	2.136 (7)	K1—O9	2.878 (7)
Sn1—C7	2.188 (7)	K1—O10	2.823 (6)
Sn1—C13	2.138 (7)	O5—C21	1.430 (9)
O1—C19	1.303 (10)	O5—C32	1.425 (9)
O2—C20	1.266 (11)	O6—C22	1.418 (12)
O3—C20	1.233 (11)	O6—C23	1.417 (11)
O3—K1	2.785 (7)	O7—C24	1.426 (10)
O4—C19	1.202 (10)	O7—C25	1.435 (11)
O4—K1	2.654 (6)	O8—C26	1.425 (11)
C1—C2	1.397 (12)	O8—C27	1.408 (11)
C1—C6	1.411 (13)	O9—C28	1.415 (11)
C2—H2	0.9500	O9—C29	1.443 (12)
C2—C3	1.422 (14)	O10—C30	1.421 (11)
C3—H3	0.9500	O10—C31	1.419 (11)
C3—C4	1.356 (15)	C21—H21A	0.9900
C4—H4	0.9500	C21—H21B	0.9900
C4—C5	1.376 (14)	C21—C22	1.493 (13)
C5—H5	0.9500	C22—H22A	0.9900
C5—C6	1.377 (13)	C22—H22B	0.9900
C6—H6	0.9500	C23—H23A	0.9900
C7—C8	1.401 (11)	C23—H23B	0.9900
C7—C12	1.389 (11)	C23—C24	1.494 (14)
C8—H8	0.9500	C24—H24A	0.9900
C8—C9	1.406 (10)	C24—H24B	0.9900
C9—H9	0.9500	C25—H25A	0.9900
C9—C10	1.405 (11)	C25—H25B	0.9900
C10—H10	0.9500	C25—C26	1.486 (13)
C10—C11	1.396 (11)	C26—H26A	0.9900
C11—H11	0.9500	C26—H26B	0.9900
C11—C12	1.407 (10)	C27—H27A	0.9900
C12—H12	0.9500	C27—H27B	0.9900
C13—C14	1.394 (15)	C27—C28	1.508 (15)
C13—C18	1.406 (14)	C28—H28A	0.9900
C14—H14	0.9500	C28—H28B	0.9900
C14—C15	1.396 (13)	C29—H29A	0.9900
C15—H15	0.9500	C29—H29B	0.9900
C15—C16	1.385 (16)	C29—C30	1.502 (14)
C16—H16	0.9500	C30—H30A	0.9900
C16—C17	1.370 (18)	C30—H30B	0.9900
C17—H17	0.9500	C31—H31A	0.9900
C17—C18	1.407 (13)	C31—H31B	0.9900
C18—H18	0.9500	C31—C32	1.506 (13)
C19—C20	1.567 (11)	C32—H32A	0.9900
K1—O5	2.976 (6)	C32—H32B	0.9900
K1—O6	2.802 (6)		
			
O1—Sn1—O2	73.4 (2)	O8—K1—O9	58.88 (19)
O1—Sn1—C1	114.0 (3)	O9—K1—O5	111.35 (18)
O1—Sn1—C7	87.6 (3)	O10—K1—O5	58.09 (15)
O1—Sn1—C13	120.4 (3)	O10—K1—O7	156.01 (19)
C1—Sn1—O2	88.5 (3)	O10—K1—O8	117.67 (18)
C1—Sn1—C7	101.9 (3)	O10—K1—O9	58.80 (19)
C1—Sn1—C13	120.5 (3)	C21—O5—K1	108.9 (4)
C7—Sn1—O2	160.8 (3)	C32—O5—K1	108.0 (4)
C13—Sn1—O2	85.5 (4)	C32—O5—C21	111.7 (6)
C13—Sn1—C7	102.5 (4)	C22—O6—K1	122.3 (5)
C19—O1—Sn1	121.9 (5)	C23—O6—K1	118.3 (6)
C20—O2—Sn1	116.1 (5)	C23—O6—C22	112.9 (7)
C20—O3—K1	117.5 (6)	C24—O7—K1	106.3 (5)
C19—O4—K1	121.4 (5)	C24—O7—C25	112.6 (7)
C2—C1—Sn1	123.1 (7)	C25—O7—K1	109.8 (5)
C2—C1—C6	118.1 (7)	C26—O8—K1	117.6 (5)
C6—C1—Sn1	118.8 (6)	C27—O8—K1	118.3 (5)
C1—C2—H2	120.5	C27—O8—C26	112.8 (6)
C1—C2—C3	119.1 (8)	C28—O9—K1	110.8 (5)
C3—C2—H2	120.5	C28—O9—C29	111.0 (7)
C2—C3—H3	119.3	C29—O9—K1	108.2 (5)
C4—C3—C2	121.4 (8)	C30—O10—K1	119.7 (5)
C4—C3—H3	119.3	C31—O10—K1	121.5 (5)
C3—C4—H4	120.2	C31—O10—C30	110.8 (7)
C3—C4—C5	119.5 (9)	O5—C21—H21A	110.0
C5—C4—H4	120.2	O5—C21—H21B	110.0
C4—C5—H5	119.5	O5—C21—C22	108.6 (7)
C4—C5—C6	121.0 (9)	H21A—C21—H21B	108.3
C6—C5—H5	119.5	C22—C21—H21A	110.0
C1—C6—H6	119.6	C22—C21—H21B	110.0
C5—C6—C1	120.8 (8)	O6—C22—C21	108.5 (7)
C5—C6—H6	119.6	O6—C22—H22A	110.0
C8—C7—Sn1	117.3 (5)	O6—C22—H22B	110.0
C12—C7—Sn1	123.7 (6)	C21—C22—H22A	110.0
C12—C7—C8	118.9 (7)	C21—C22—H22B	110.0
C7—C8—H8	119.5	H22A—C22—H22B	108.4
C7—C8—C9	121.1 (7)	O6—C23—H23A	110.0
C9—C8—H8	119.5	O6—C23—H23B	110.0
C8—C9—H9	120.3	O6—C23—C24	108.5 (8)
C10—C9—C8	119.3 (7)	H23A—C23—H23B	108.4
C10—C9—H9	120.3	C24—C23—H23A	110.0
C9—C10—H10	120.1	C24—C23—H23B	110.0
C11—C10—C9	119.8 (7)	O7—C24—C23	109.1 (7)
C11—C10—H10	120.1	O7—C24—H24A	109.9
C10—C11—H11	120.0	O7—C24—H24B	109.9
C10—C11—C12	120.0 (7)	C23—C24—H24A	109.9
C12—C11—H11	120.0	C23—C24—H24B	109.9
C7—C12—C11	120.9 (8)	H24A—C24—H24B	108.3
C7—C12—H12	119.6	O7—C25—H25A	109.9
C11—C12—H12	119.6	O7—C25—H25B	109.9
C14—C13—Sn1	118.1 (7)	O7—C25—C26	108.9 (7)
C14—C13—C18	119.9 (7)	H25A—C25—H25B	108.3
C18—C13—Sn1	122.0 (7)	C26—C25—H25A	109.9
C13—C14—H14	119.8	C26—C25—H25B	109.9
C13—C14—C15	120.5 (8)	O8—C26—C25	108.3 (7)
C15—C14—H14	119.8	O8—C26—H26A	110.0
C14—C15—H15	120.1	O8—C26—H26B	110.0
C16—C15—C14	119.7 (9)	C25—C26—H26A	110.0
C16—C15—H15	120.1	C25—C26—H26B	110.0
C15—C16—H16	120.0	H26A—C26—H26B	108.4
C17—C16—C15	120.0 (8)	O8—C27—H27A	109.9
C17—C16—H16	120.0	O8—C27—H27B	109.9
C16—C17—H17	119.1	O8—C27—C28	108.8 (8)
C16—C17—C18	121.7 (9)	H27A—C27—H27B	108.3
C18—C17—H17	119.1	C28—C27—H27A	109.9
C13—C18—C17	118.1 (9)	C28—C27—H27B	109.9
C13—C18—H18	121.0	O9—C28—C27	107.8 (8)
C17—C18—H18	121.0	O9—C28—H28A	110.1
O1—C19—C20	114.2 (7)	O9—C28—H28B	110.1
O4—C19—O1	124.3 (8)	C27—C28—H28A	110.1
O4—C19—C20	121.5 (8)	C27—C28—H28B	110.1
O2—C20—C19	113.8 (7)	H28A—C28—H28B	108.5
O3—C20—O2	128.3 (8)	O9—C29—H29A	110.2
O3—C20—C19	117.9 (8)	O9—C29—H29B	110.2
O3—K1—O5	128.47 (19)	O9—C29—C30	107.7 (8)
O3—K1—O6	99.3 (2)	H29A—C29—H29B	108.5
O3—K1—O7	83.7 (2)	C30—C29—H29A	110.2
O3—K1—O8	77.22 (19)	C30—C29—H29B	110.2
O3—K1—O9	104.4 (2)	O10—C30—C29	107.5 (7)
O3—K1—O10	119.8 (2)	O10—C30—H30A	110.2
O4—K1—O3	61.30 (19)	O10—C30—H30B	110.2
O4—K1—O5	68.16 (17)	C29—C30—H30A	110.2
O4—K1—O6	75.94 (18)	C29—C30—H30B	110.2
O4—K1—O7	117.6 (2)	H30A—C30—H30B	108.5
O4—K1—O8	138.06 (19)	O10—C31—H31A	110.0
O4—K1—O9	123.7 (2)	O10—C31—H31B	110.0
O4—K1—O10	81.05 (18)	O10—C31—C32	108.5 (7)
O6—K1—O5	57.71 (16)	H31A—C31—H31B	108.4
O6—K1—O7	59.86 (18)	C32—C31—H31A	110.0
O6—K1—O8	119.38 (18)	C32—C31—H31B	110.0
O6—K1—O9	154.6 (2)	O5—C32—C31	109.2 (6)
O6—K1—O10	115.80 (18)	O5—C32—H32A	109.8
O7—K1—O5	112.53 (18)	O5—C32—H32B	109.8
O7—K1—O9	113.74 (19)	C31—C32—H32A	109.8
O8—K1—O5	153.77 (17)	C31—C32—H32B	109.8
O8—K1—O7	59.61 (19)	H32A—C32—H32B	108.3
			
Sn1—O1—C19—O4	−171.7 (6)	K1—O3—C20—C19	6.8 (13)
Sn1—O1—C19—C20	9.4 (10)	K1—O4—C19—O1	179.9 (6)
Sn1—O2—C20—O3	179.5 (10)	K1—O4—C19—C20	−1.3 (11)
Sn1—O2—C20—C19	0.0 (11)	K1—O5—C21—C22	−59.3 (7)
Sn1—C1—C2—C3	−178.2 (6)	K1—O5—C32—C31	60.9 (7)
Sn1—C1—C6—C5	179.2 (7)	K1—O6—C22—C21	−35.7 (10)
Sn1—C7—C8—C9	178.1 (5)	K1—O6—C23—C24	29.9 (9)
Sn1—C7—C12—C11	−178.2 (7)	K1—O7—C24—C23	65.7 (7)
Sn1—C13—C14—C15	−176.6 (7)	K1—O7—C25—C26	−60.8 (8)
Sn1—C13—C18—C17	176.8 (6)	K1—O8—C26—C25	−37.5 (9)
O1—C19—C20—O2	−5.7 (12)	K1—O8—C27—C28	38.8 (9)
O1—C19—C20—O3	174.8 (9)	K1—O9—C28—C27	59.3 (8)
O4—C19—C20—O2	175.4 (9)	K1—O9—C29—C30	−64.4 (8)
O4—C19—C20—O3	−4.2 (14)	K1—O10—C30—C29	−34.4 (9)
C1—C2—C3—C4	−0.2 (13)	K1—O10—C31—C32	33.9 (8)
C2—C1—C6—C5	−0.1 (13)	O5—C21—C22—O6	63.9 (9)
C2—C3—C4—C5	−1.7 (14)	O6—C23—C24—O7	−65.8 (9)
C3—C4—C5—C6	2.7 (15)	O7—C25—C26—O8	66.3 (9)
C4—C5—C6—C1	−1.8 (14)	O8—C27—C28—O9	−66.0 (10)
C6—C1—C2—C3	1.1 (12)	O9—C29—C30—O10	66.4 (9)
C7—C8—C9—C10	−1.1 (11)	O10—C31—C32—O5	−64.5 (8)
C8—C7—C12—C11	−1.7 (13)	C21—O5—C32—C31	−179.4 (7)
C8—C9—C10—C11	1.1 (11)	C22—O6—C23—C24	−177.8 (7)
C9—C10—C11—C12	−1.5 (13)	C23—O6—C22—C21	173.2 (8)
C10—C11—C12—C7	1.8 (14)	C24—O7—C25—C26	−179.0 (7)
C12—C7—C8—C9	1.4 (12)	C25—O7—C24—C23	−174.0 (7)
C13—C14—C15—C16	−0.7 (14)	C26—O8—C27—C28	−178.2 (8)
C14—C13—C18—C17	−1.0 (12)	C27—O8—C26—C25	179.2 (8)
C14—C15—C16—C17	−0.1 (14)	C28—O9—C29—C30	173.8 (7)
C15—C16—C17—C18	0.3 (14)	C29—O9—C28—C27	179.6 (8)
C16—C17—C18—C13	0.2 (13)	C30—O10—C31—C32	−177.4 (7)
C18—C13—C14—C15	1.3 (13)	C31—O10—C30—C29	176.3 (7)
K1—O3—C20—O2	−172.6 (9)	C32—O5—C21—C22	−178.5 (7)

### (1,4,7,10,13,16-Hexaoxacyclooctadecane-1κ6O)(µ-oxalato-1κ2O1,O2:2κ2O1',O2')triphenyl-2κ3C-potassium(I)tin(IV) Hydrogen-bond geometry (Å, º)

**Table:**

*D*—H···*A*	*D*—H	H···*A*	*D*···*A*	*D*—H···*A*
C4—H4···O1^i^	0.95	2.42	3.360 (11)	172
C16—H16···O1^ii^	0.95	2.41	3.332 (9)	165

Symmetry codes: (i) *x*+1, *y*, *z*; (ii) *x*, *y*, *z*+1.
